# Searching for potential acetylcholinesterase inhibitors: a combined approach of multi-step similarity search, machine learning and molecular dynamics simulations

**DOI:** 10.1098/rsos.240546

**Published:** 2024-10-02

**Authors:** Quynh Mai Thai, Minh Quan Pham, Phuong-Thao Tran, Trung Hai Nguyen, Son Tung Ngo

**Affiliations:** ^1^ Laboratory of Biophysics, Institute for Advanced Study in Technology, Ton Duc Thang University, Ho Chi Minh City 72915, Vietnam; ^2^ Faculty of Pharmacy, Ton Duc Thang University, Ho Chi Minh City 72915, Vietnam; ^3^ Institute of Natural Products Chemistry, Vietnam Academy of Science and Technology, Hanoi 11307, Vietnam; ^4^ Graduate University of Science and Technology, Vietnam Academy of Science and Technology, Hanoi 11307, Vietnam; ^5^ Hanoi University of Pharmacy, 13-15 Le Thanh Tong, Hanoi 100000, Vietnam

**Keywords:** AChE, binding free energy, machine learning, MD simulations

## Abstract

Targeting acetylcholinesterase is one of the most important strategies for developing therapeutics against Alzheimer’s disease. In this work, we have employed a new approach that combines machine learning models, a multi-step similarity search of the PubChem library and molecular dynamics simulations to investigate potential inhibitors for acetylcholinesterase. Our search strategy has been shown to significantly enrich the set of compounds with strong predicted binding affinity to acetylcholinesterase. Both machine learning prediction and binding free energy calculation, based on linear interaction energy, suggest that the compound CID54414454 would bind strongly to acetylcholinesterase and hence is a promising inhibitor.

## 1. Introduction

Alzheimer’s disease (AD) is the most common type of dementia and causes the deterioration of memory and cognitive functions in millions of senior adults worldwide [1,2]. In addition, there has been a sharp rise in the number of patients in recent years [3]. Unfortunately, despite considerable efforts by the scientific community [4–8], effective treatment for AD remains elusive [1,9].

According to the cholinergic hypothesis [10,11], the mechanism of AD is associated with acetylcholinesterase (AChE), an enzyme that hydrolyses acetylcholine (ACh), a neurotransmitter. The enzyme AChE has been recognized as a primary target for the development of drugs to treat AD [12,13]. Inhibiting AChE in cholinergic neurons may hinder synaptic depression and prevent the breakdown of ACh. There are currently several US Food and Drug Administration approved commercial drugs such as donepezil [12], galantamine [14] and rivastigmine [15]. Nevertheless, these drugs induce numerous adverse side effects. Consequently, the investigation of new inhibitors targeting AChE continues to attract significant interest among researchers [16–18].

Computational approaches have been increasingly employed to search for potential inhibitors that can bind with a strong affinity to a protein target and inhibit its biological activity [19–25]. Molecular docking [26] has been commonly used for initial screening of large databases of compounds to reduce to a short list of potential compounds. More computationally demanding methods, such as free energy perturbation [27,28], fast pulling of ligands [29], and linear interaction energy (LIE) [30,31], are used for more accurate prediction of binding free energy for compounds in the shortlist. Recently, machine learning (ML) methods have shown great potential in fast and accurate screening of potential drugs [32–34]. One promising application of ML methods is in initial screening of large numbers of ligands for potential inhibitors [35–37]. The prediction from ML is then validated through molecular dynamics (MD) simulations and physics-based free energy calculations [24,25,37].

In this study, we apply the ML method in combination with a multi-step similarity search of the PubChem database to screen for potential AChE inhibitors. The best-predicted compound is then validated by MD simulations and binding free energy calculation based on LIE.

## 2. Material and methods

**Figure 1.**
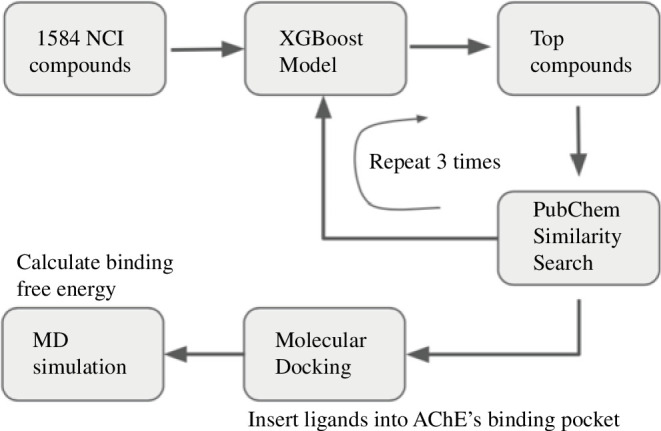
Computational scheme for screening of potential acetylcholinesterase (AChE) inhibitors. MD, molecular docking.

Our computational strategy to screen for potential AChE inhibitors is depicted in figure 1. First, a ML model was employed to predict binding free energy for 1584 National Cancer Institute (NCI) compounds, of which the top compounds having the strongest predicted binding free energy were selected. Next, multiple rounds of similarity search from the PubChem library were combined with ML prediction to enrich the set of top compounds. These top compounds were then docked into the AChE binding pocket to create initial structures for MD simulations. Finally, data from MD simulations were used to carry out binding free energy calculations based on the LIE method.

### 2.1. Data collection

The SMILES strings of 1584 compounds were downloaded from the NCI Diversity Set VII [38]. Structures of human AChEs in complex with 10 inhibitors were downloaded from the Protein Data Bank with the identifiers 6CQV [39], 4M0E, 4M0F [40], 4EY5, 4EY6, 4EY7 [41], 6O50 [42], 7D9Q, 7D9O and 7D9P [43].

### 2.2. Training of machine learning models

Regression models were trained in our previous work (QM Thai, TH Nguyen, GB Lenon, HTT Phung Horng, PT Thai, Nguyen, Lenon, Phung JT, Tran, submitted). Here, we provide an overview of the model training process. SMILES and experimental binding constants (*K*_i_) for 1426 compounds were collected from the binding database [44,45]. *K*_i_ was converted into binding free energy using the formula $\Delta G=RTlnK_{i}$ , where *R* is gas constant and *T* = 298 K is the absolute temperature. A total of 1064 compounds were randomly selected for training and the rest (362) were used for testing the models.

We trained four ML models: linear regression (LR), random forest (RF), extreme gradient boosting (XGBoost) [46] and graph-convolutional networks (Graph-Conv) [47]. Features for LR, RF and XGBoost models were calculated using the RDKitDescriptors toolkit, implemented in DeepChem [48]. These features are descriptors describing physicochemical properties, such as molecular weight, number of valence electrons, numbers of hydrogen bond (HB) donors and acceptors, and polar surface area. GraphConv is a deep learning approach that can learn features on the fly and therefore, removes the need to manually extract features. The model performance on the test set of 361 compounds was measured by root mean square error (RMSE) and the two correlation coefficients, Pearson’s *R* and Spearman’s ρ . XGBoost gave the best test performance, with lowest RMSE = 1.357 ± 0.096 kcal mol^−1^ ( 1 kcal mol^−1^ = 4.184 kJ mol^−1^) and highest correlation coefficients, Pearson’s R=0.813±0.027 and Spearman’s ρ=0.808±0.026 . The trained model showed significant improvement compared with the one from our previous work [25], which was trained on fewer data. Electronic supplementary material, figure S1 shows a comparison between experimental and ML-predicted binding free energy for the test compounds.

### 2.3. Prediction of binding free energy using machine learning model

The best XGBoost model was employed to predict binding affinity for 1584 NCI compounds. The top 17 compounds with predicted binding free energy lower than −10 kcal mol^−1^ (electronic supplementary material, table S1) were selected for the subsequent similarity search of the PubChem library.

### 2.4. Similarity search of PubChem library

A two-round similarity search was performed. In the first round, compounds similar to at least one of those in the list of the top 17 compounds (electronic supplementary material, table S1) were searched from the PubChem library [49], resulting in 5393 unique compounds. Binding free energy was predicted for these newly identified compounds using the XGBoost model. In the second round, another list of the top nine compounds with the lowest predicted binding free energy were chosen (electronic supplementary material, table S2). A similarity search in PubChem for this list resulted in 1768 unique compounds for which ML prediction of binding free energy was also made.

### 2.5. Molecular docking

AutoDock Vina [50] with modified empirical parameters [51] was employed to dock the ligand with lowest binding free energy by XGBoost into the binding site of AChE, whose crystal structure was downloaded from the Protein Data Bank with identifier 4M0E [40]. The receptor and ligands were parameterized using a force field provided by AutoDockTools. The centre of the docking grid was chosen as the centre of mass of dihydrotanshinone I bound to AChE in the crystal structure. The docking grid size was chosen as 26 × 26 × 26 Å^3^. Structures with the lowest energy from docking were captured for further analysis through MD simulations.

### 2.6. Molecular dynamics simulations

Atomistic simulations in aqueous solution were carried out for 11 AChE–inhibitor complexes, including 10 crystal structures with PDB identifiers 6CQV, 4M0E, 4M0F, 4EY5, 4EY6, 4EY7, 6O50, 7D9Q, 7D9O and 7D9P, and the docked structure best candidate inhibitor predicted by ML to AChE is 4M0E. Simulations were also performed for 11 free ligands in water boxes. GROMACS 2019.6 [52] was employed to sample conformational space of the receptor–ligand complexes and ligands in aqueous solution. The receptor and ions were parameterized by the Amber99SB-iLDN force field [53] ,whereas the TIP3P water model [54] was used for water molecules. The bonded and Lennard Jones parameters of ligands were taken from the general Amber force field (GAFF) [55], whereas the point charges were fitted using the restrained electrostatic potential method [55] implemented in the AmberTools18 [56] and ACPYPE packages [57]. The electrostatic potential grids used for fitting point charges were obtained from density functional theory (DFT) quantum mechanical calculations using the double hybrid Mp2 (Møller–Plesset second-order perturbation theory) functional, basis set 6-31G(d,p), and implicit solvent ( ε=78.4 ). The AChE–ligand complexes were placed in a box with dodecahedral periodic boundary conditions, ensuring a minimum separation of 16.0 Å from the complex to the box edge. The volume of the box was approximately 937.53 nm³ and the total number of atoms is roughly 92 000 for AChE–ligand complexes. For the free ligands, the simulation box has a volume of 58.60 nm³ and contains 5700 atoms in total.

First, the complexes underwent energy minimization using the steepest descent technique. Then the minimized systems were subjected to a relaxation process through 100 ps of NVT and NPT simulations, during which a slight harmonic force was applied to restrain the Cα atoms. The final snapshot from the NPT simulations served as the initial configuration for the subsequent unbiased MD simulations. These MD simulations were carried out for a duration of 50 ns each. To guarantee adequate sampling of the complexes’ conformations, MD simulations were repeated four times using different random number seeds.

### 2.7. Linear interaction energy calculation

Binding free energy calculations based on MD simulations have been widely used to study protein–ligand binding [58,59]. In this work, the AChE–ligand binding free energy $\Delta G_{LIE}$ was estimated using the LIE method [30,31]. According to this method, the binding free energy is expressed as a linear function of the differences in mean interaction energies between the bound and unbound states. In particular, $\Delta G_{LIE}$ is written as

,$$\Delta G_{LIE}=\alpha \left(<V_{l\text{-}s}^{vdw}{>}_{b}-<V_{l\text{-}s}^{vdw}{>}_{u}\right)\;+\beta \left(<V_{l\text{-}s}^{cou}{>}_{b}-<V_{l\text{-}s}^{cou}{>}_{u}\right)+\gamma$$

where vdw and cou indicate van der Waals and Coulomb terms, respectively. The subscript l-s stands for ligand-surrounding and indicates the interaction energy between ligand and the surrounding environment. The subscripts b and u stand for bound and unbound states, respectively. MD trajectories for the bound AChE–ligand complexes in water were used to calculate the interaction energy in the bound state whereas the MD trajectories for the free ligand in water were used to calculate the interaction energy in the unbound state. Moreover, the shallow binding cleft of AChE bears a resemblance to the active site of SARS-CoV-2 M^pro^. This suggests potential similarities in the physical behaviour of these receptors. Consequently, the empirical parameters α=0.288 , β=-0.049 and γ=-5.88 from the SARS-SARS-CoV-2 M^pro^ system [60] are suggested for calculating the ligand-binding affinity of the AChE+inhibitor.

### 2.8. Analysis tools

The root mean square deviation (RMSD) was calculated using the GROMACS tool ‘gmx rms’ [52]. The program ‘gmx hbond’ was used for analysing the intermolecular hydrogen bonds (HBs). HBs between the residual AChE and ligand were calculated when the angle between acceptor–hydrogen–donor is ≥135° and the distance between acceptor and donor is ≤3.5 Å. The intermolecular side chain contact (SC) between the ligand and the residual AChE was calculated based on the distance between their non-hydrogen atoms, which must be ≤4.5 Å. In addition, pharmacokinetic parameters and toxicity of potential compounds were predicted via PreADMET Web app [61]. This method has been shown to provide predictions that are highly correlated with experimental data.

## 3. Results and discussion

### 3.1. Machine learning prediction of binding free energy

**Figure 2.**
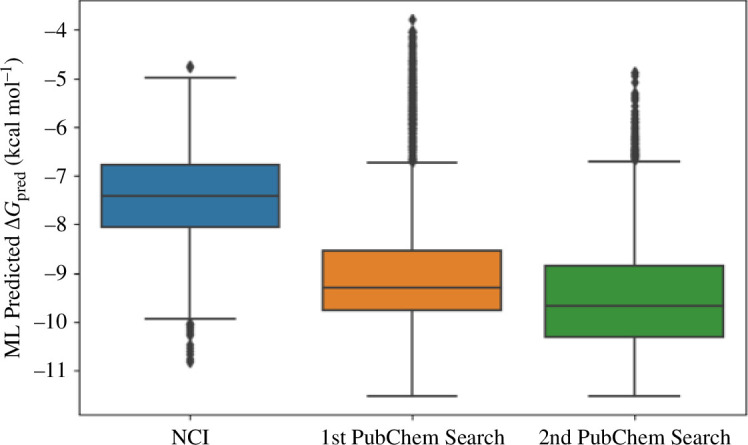
Distribution of the binding free energy predicted by the XGBoost model for ligands in the NCI library and for ligands that resulted from the first (1st PubChem Search) and second (2nd PubChem Search) rounds of similarity search of the PubChem library.

The best ML model, XGBoost, was used to predict the binding free energy for 1584 compounds in the NCI library, the 5393 PubChem compounds that resulted from the first similarity search round and 1768 PubChem compounds in the second similarity search round. Figure 2 shows the distributions of the predicted binding free energy, $\Delta G_{pred}$ , where we can see clearly that our multi-step similarity search of the PubChem library helped enrich the ligand set with more and more ligands having strong predicted binding affinity to AChE. In particular, the predicted binding free energy for compounds in the NCI compound set has the interquartile range −8.05 to −6.77 kcal mol^−1^ and a median value of −7.42 kcal mol^−1^. After the first round of similarity search in the PubChem library, the compound set adopted a significantly lower interquartile range of −9.77 to −8.54 kcal mol^−1^ with a median value of −9.30 kcal mol^−1^. The second similarity search shifts the distribution of predicted binding free energy further down to an interquartile range of −10.30 to −8.85 kcal mol^−1^ with the median at −9.65 kcal mol^−1^. This apparently shows that our similarity search procedure from the PubChem library is very effective in finding compounds having strong predicted binding free energy to AChE. The predicted strongest-binding compound has the PubChem CID 54414454 and XGBoost binding free energy of −11.51 kcal mol^−1^. Subsequent MD simulation and binding free energy estimation of this compound would provide important insights into the structural and energetic aspects of the binding process with atomistic details.

### 3.2. Molecular dynamics simulations

**Figure 3.**
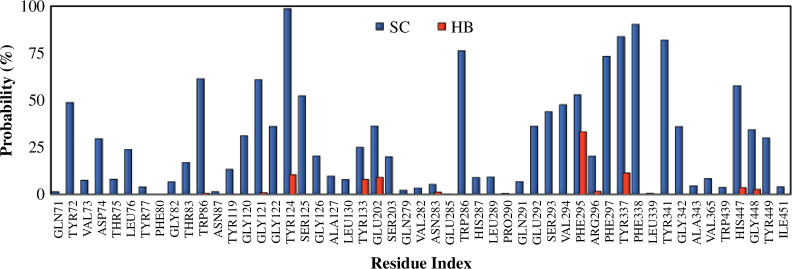
Probability for the compound 54414454 to form side chain (SC) and hydrogen bond (HB) contacts with residues of acetylcholinesterase. The probability was estimated as the fraction of time the contacts were formed over the total simulation time.

**Figure 4.**
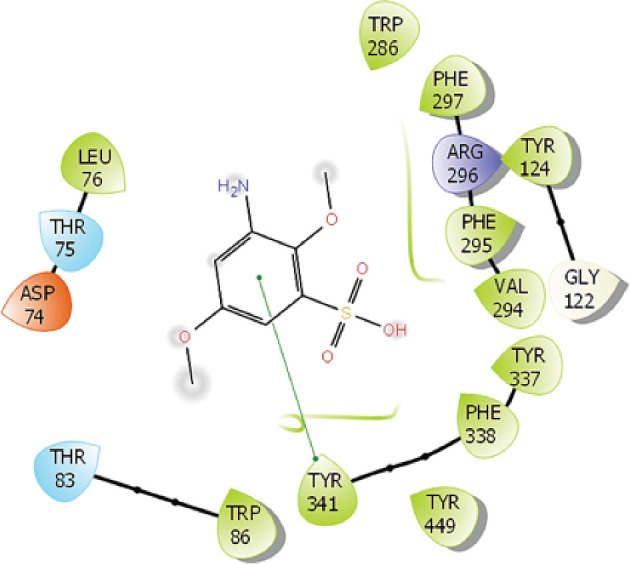
2-Dimensional presentation of binding pose of 54414454 with acetylcholinesterase using a snapshot of molecular docking simulation. The diagram was prepared by using Maestro software.

Two independent MD simulations, each having a 50 ns trajectory, were performed for the complex between compound 54414454 and AChE. The RMSD plot over simulation time (electronic supplementary material, table S3) shows that the system has reached equilibrium. Important insights into the binding process can be gained by investigating how the ligand interacts with the surrounding amino acids in the binding pocket of AChE. Figure 3 shows the probability (fraction of time) of compound 54414454 making side chain (hydrophobic) contacts as well as HBs with residues of AChE. While the compound makes fewer HBs with AChE, its binding to AChE is driven mostly by hydrophobic interaction. In particular, it is found that the four residues TYR124, GLU202, PHE295 and TYR337 might play important roles in the binding of compound 54414454 since they contribute to both HB contacts and significant side chain contacts (figure 4).

### 3.3. Binding free energy calculations

**Figure 5.**
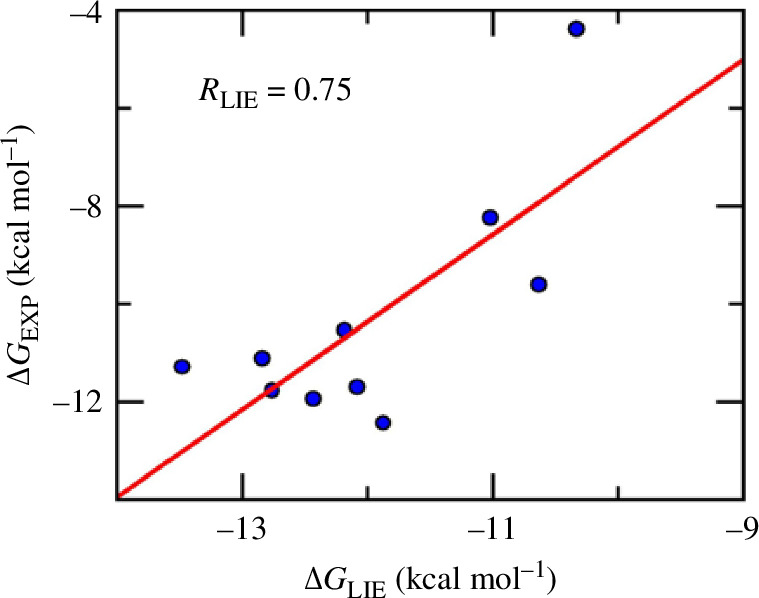
Comparison of binding free energy between calculation based on linear interaction energy (LIE) method and experiment for 10 acetycholinesterase–ligand complexes.

To validate our binding free energy calculation approach based on the LIE method, we calculated binding free energy $\Delta G_{LIE}$ for 10 experimentally available AChE–ligand complexes for which both crystal structures and IC50 data are available. Table 1 shows a comparison between our calculation results and experiment. The calculated $\Delta G_{LIE}$ is highly correlated with the experimental values (figure 5), with the correlation coefficient Pearson’s *R* = 0.75. The RMSE of our calculated result (with respect to experimental $\Delta G_{EXP}$ ) is not too high (RMSE = 2.37 kcal mol^−1^). Therefore, the linear interaction energy approach can estimate binding free energy for AChE–ligand in good agreement with experiment. We then applied this approach for the best candidate compound CID54414454 predicted by the XGBoost model and the result is shown in the last row of table 1. The LIE gives an estimate of binding free energy for CID54414454 that is a little bit weaker than the 10 experimentally available complexes. A plausible explanation is that the LIE calculations for the 10 experimentally available complexes were based on native poses of the ligands in which the ligand already adopted the most favourable binding structures. On the other hand, the LIE calculation for compound CID54414454 was based on the docked pose, which may not be the most energetically favourable pose and the subsequent MD simulations were not able to relax the complex to the right binding pose. Nevertheless, the newly found ligand is still a good binder to AChE and can serve as a basis for further AD drug development targeting AChE.

**Table 1.** Binding free energy calculation results using linear interaction energy (LIE) method for 10 experimentally available complexes and the best candidate compound CID54414454 predicted by our machine learning model.

PDB ID	resolution (Å)	ligand	IC_50_ (nm)	Δ*G*_EXP_ (kcal mol^−1^)^a^	$\Delta G_{LIE}$ (kcal mol^−1^)
6CQV [39]	2.60	HI6	636 000	−4.39	−10.33 ± 0.36
4M0E [40]	2.00	dihydrotanshinone I	1000	−8.24	−11.02 ± 0.15
4EY6 [41]	2.40	galantamine	100	−9.61	−10.63 ± 0.14
4EY5 [41]	2.30	huperzine A	21	−10.54	−12.18 ± 0.19
6O50 [42]	2.35	EBW	8	−11.11	−12.84 ± 0.40
4M0F [40]	2.30	territrem B	6	−11.29	−13.48 ± 0.54
7D9Q [43]	2.66	H1R	3	−11.70	−12.08 ± 0.82
7D9O [43]	2.45	H0L	3	−11.77	−12.76 ± 0.20
4EY7 [41]	2.35	donepezil	2	−11.94	−12.43 ± 0.72
7D9P [43]	2.85	H0R	0.86	−12.44	−11.87 ± 0.61
		CID54414454			−10.45 ± 0.26

^a^ The experimental values of $\Delta G_{EXP}$ are estimated from the inhibition constants IC_50_ via the formula Δ*G* = *RT*ln(IC_50_). The computed error of ΔG_LIE_ is the s.e. of the mean over two independent trajectories.

## 4. Conclusion

We have applied ML and MD simulations to search for potential AChE inhibitors. Our combined approach of using an ML model and multi-step similarity search of the PubChem library has substantially enriched the set of compounds with strong predicted binding free energy to AChE. The best compound, CID54414454, that resulted from the search was further investigated using MD simulations to gain molecular insights into the binding process. Important residues such as TYR124, GLU202, PHE295 and TYR337 were suggested to play an important role in binding of CID54414454 to AChE. Our LIE binding free energy calculations for 10 experimentally available AChE–ligand complexes showed good correlation with experiment. The LIE calculation for CID54414454 suggested that the compound would be a promising inhibitor for AChE and could serve as the basis for further drug development targeting AChE.

## Data Availability

Relevant data necessary to reproduce all results in the paper are provided within the main text and in the online supplementary material that accompanies this article [62]. Data and relevant code for this research work are stored in GitHub at [63] and have been archived within the Zenodo repository [64].
